# Contemplating the necessity of surgical treatment for posterior lenticonus: a case report

**DOI:** 10.1186/s12886-023-03042-9

**Published:** 2023-06-20

**Authors:** Ming-zhi Lu, Wen-li Cao, Yi-qiao Xing

**Affiliations:** Department of Ophthalmology, Wuhan Aier eye hospital, Wuhan University, Wuhan, Hubei China

**Keywords:** Lenticonus, Case report, Lens and anterior segment optical coherence

## Abstract

**Background:**

Posterior lenticonus is an uncommon congenital abnormality that causes a progressive, localized spherical or conical bulging of the posterior capsular membrane, resulting in an abnormal shape of the lens.

**Case presentation:**

A 13-year-old girl presented with ametropia in both eyes. After mydriasis, examination revealed an oval bubble-shaped alteration with a distinct boundary above the temporal region on the center of the posterior capsule of her left lens. The subcortical region surrounding the alteration appeared feathery and turbid. The patient had no history of trauma or family history of visual impairment. Systemic investigations were normal. A thorough eye examination was performed, which included optometry, ultrasound biomicroscopy, ocular B-Scan, and anterior segment optical coherence, to assess the disease. The patient was diagnosed with posterior lenticonus in the left eye, as well as ametropia and anisometropia in both eyes. Conservative treatment was initiated since the patient’s current best corrected visual acuity was good, and regular monitoring of the condition’s progression was scheduled.

**Conclusions:**

This case report presents a rare instance of posterior lenticonus. The findings of this report raise new considerations regarding the necessity of surgical intervention for this condition.

## Background

Posterior lenticonus is a rare congenital abnormality that results in a localized and progressive bulge in either a spherical or conical shape found in the posterior capsular membrane of the lens. This patient case report thoroughly documents the adjuvant examination and presents a rare instance of posterior lenticonus in a single eye. We present this case report with the aim of raising awareness among ophthalmologists about the existence of congenital lens anomalies that can be accurately detected, closely monitored, and potentially treated surgically, if necessary. However, this particular case serves as a reminder that not all such conditions require surgery. While there are no established guidelines to indicate the clear indications for surgery, we can use this case as a reference to determine whether surgical intervention is needed.

We would like to emphasize that we conducted a series of comprehensive auxiliary examinations to evaluate the patient’s condition, including optometry examination, Anterior Segment Optical Coherence Tomography (AS-OCT), Under ultrasound biomicroscopy (UBM), B-scan, and fundus photography. In the process, we found that AS-OCT Tomography was particularly useful in diagnosis, outperforming other auxiliary examinations. Hence, we believe that AS-OCT Tomography is a superior tool for diagnosis, and we recommend its application to other similar cases.

## Case presentation

**Fig. 1:**
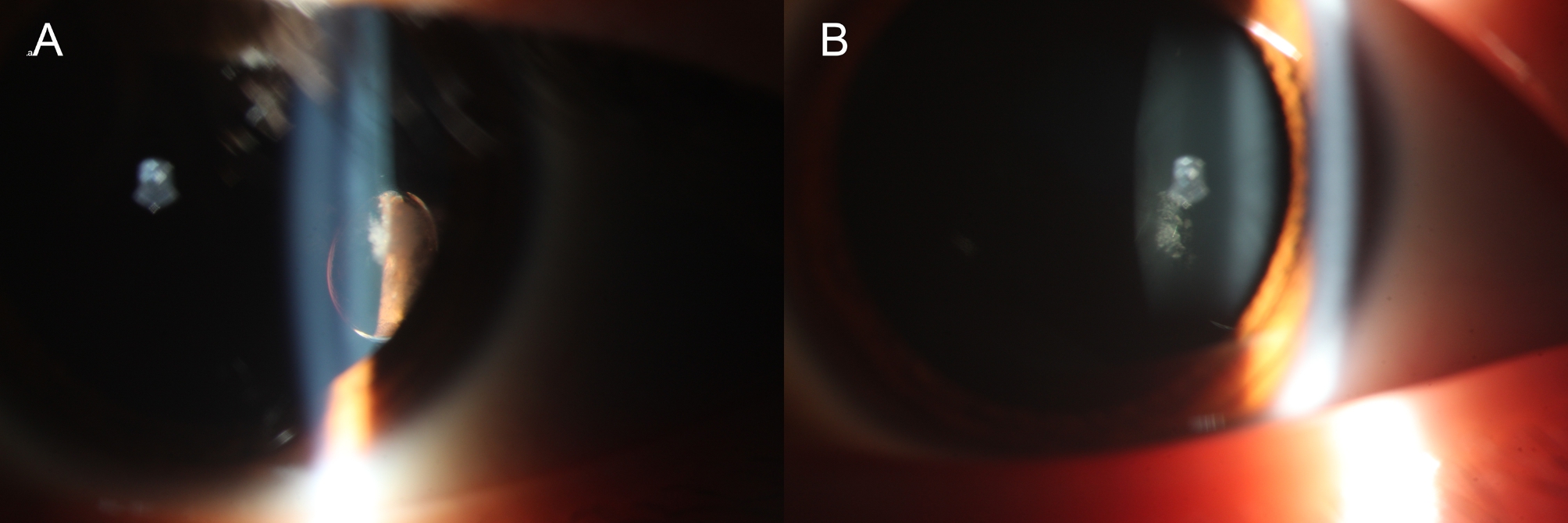
Slit lamp photography of the patient’s anterior segment

The patient is a 13-year-old female who, five years ago, experienced impaired vision without any accompanying symptoms such as eye pain, redness, photophobia, tears, or other discomfort. Her medical history revealed no previous incidents of trauma, and there was no known hereditary visual impairment in the family. The results of other systemic tests conducted were normal and unremarkable. During the ophthalmic examination, the patient exhibited a visual acuity of 20/40 in the right eye and 20/32 in the left eye. The intraocular pressure was within the normal range, and no strabismus was observed, with normal eye movement. The anterior segment examination under slit-lamp revealed normal findings. Subsequently, 1% tropicamide eye drops were used to dilate the pupils. The right lens displayed normal findings, while the left lens appeared normal in size with a well-circumscribed, oval-shaped vesicular alteration above the temporoparietal center of the posterior capsule, accompanied by a feather-like opacity in the nearby posterior subcapsular cortex (Fig. 1). Other lens were transparent, with a normal form and position of the suspensory ligament.

**Fig. 2:**
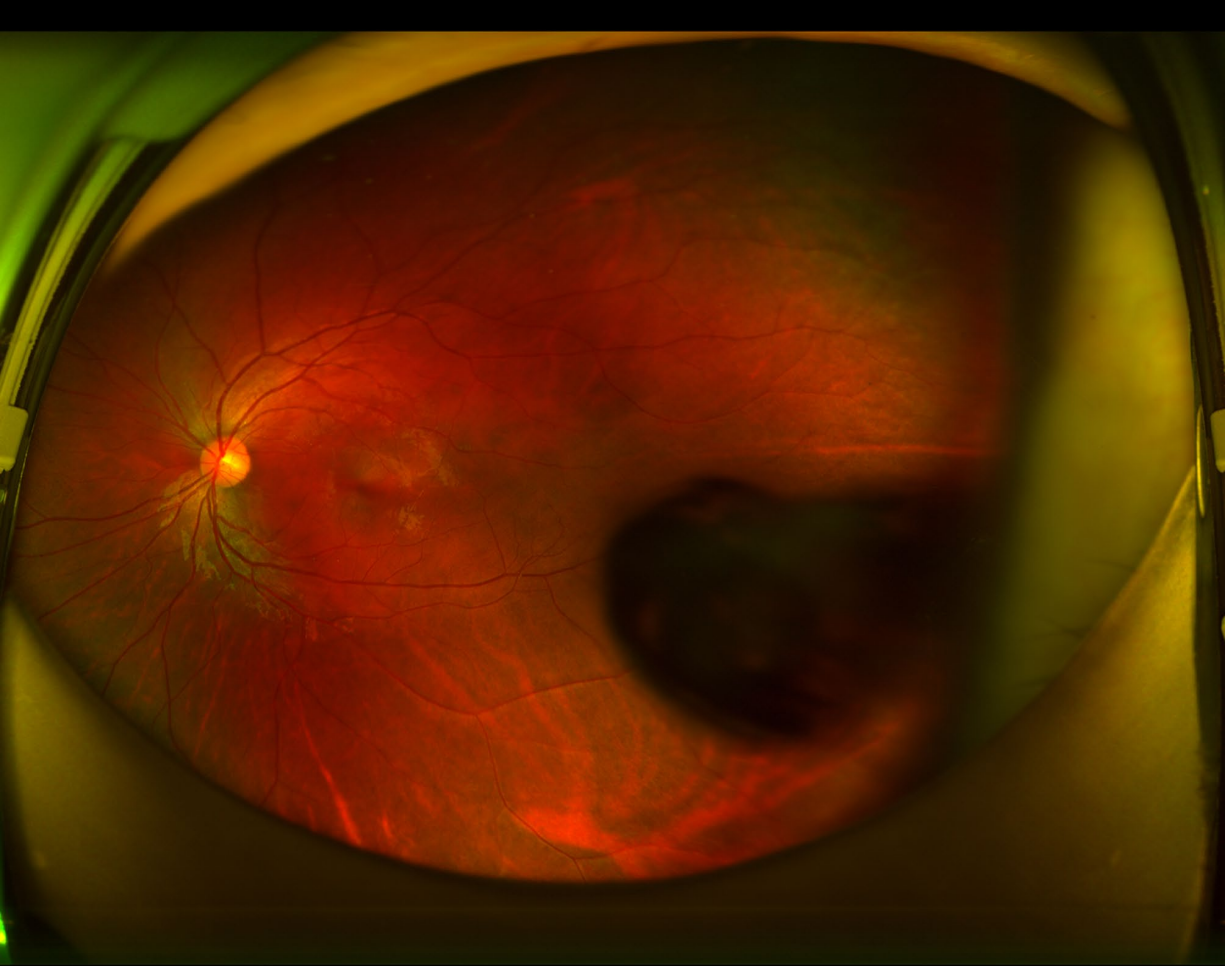
Fundus photography of the patient’s left eye

**Fig. 3:**
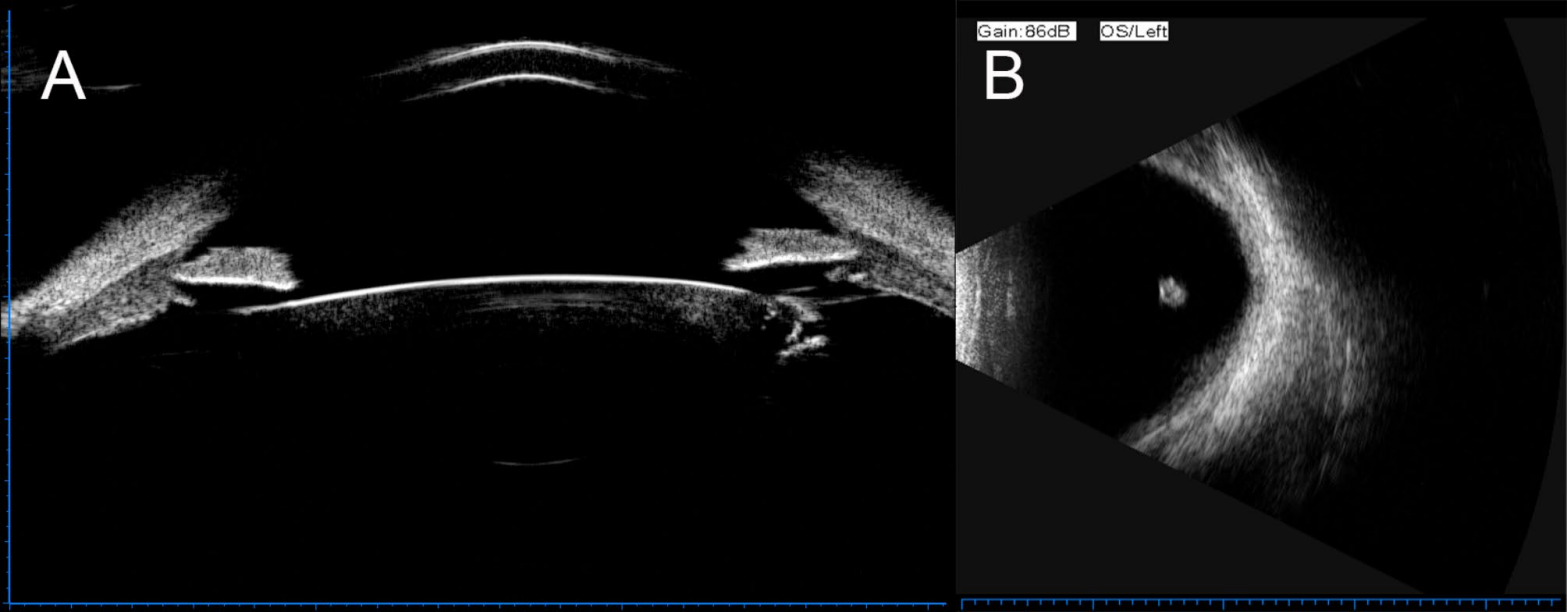
Ultrasound biomicroscopy and Ocular B-Scan of the patient’s left eye

**Fig. 4:**
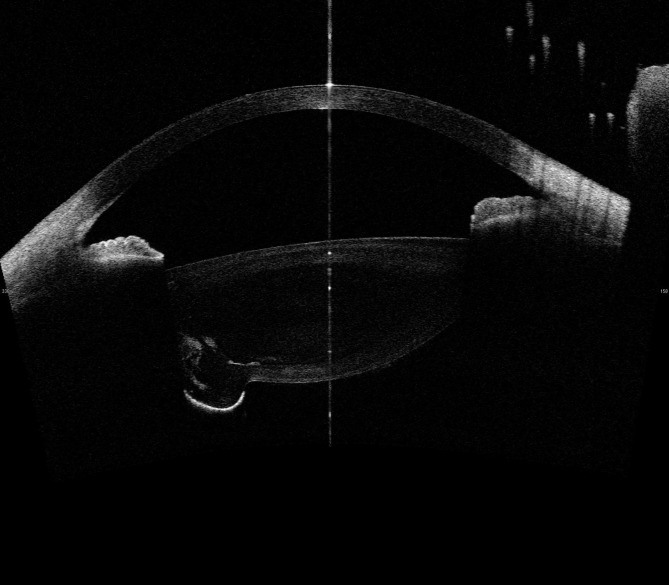
Anterior Segment Optical Coherence of patient’s left eye

Fundus exam appeared normal. However, the fundus image appeared distorted when posterior lenticonus lens changes were visible (Fig. 2). -UBM, the temporal crystal of the left eye exhibited an abnormally strong echo (Fig. 3A), while the vitreous cavity showed large, fixed hyperechoic masses seen through ocular B-scan (Fig. 3B). Cystic hyperreflection was visible behind the left eye lens according to AS-OCT (Fig. 4). The patient currently suffers from posterior lenticonus in the left eye, as well as ametropia and anisometropia in both eyes. The best-corrected visual acuity was 20/20 OD and 20/25 OS, with a refractive status of -1.25/−2.25 × 5 OD and + 4.75/−4.50 × 165 OS. The patient is undergoing temporary conservative therapy with eyewear and regular monitoring, as she currently has good vision correction with no other discernible symptoms.

## Discussion and conclusions

Posterior lenticonus is a rare congenital eye condition with a prevalence of 1 in 100,000. In 95% of cases, it is sporadic and monocular [1, 2]. It is uncommon for both eyes to be affected by a hereditary form of the disease, which can be inherited through autosomal dominant or X chromosome-linked inheritance [3, 4]. Patients with posterior lenticonus typically do not exhibit other associated congenital ocular or systemic abnormalities. The bulge on the localized posterior capsule of the lens appears more prominently on the nasal side and is generally found in the paracentral or central regions [5].

Posterior lenticonus is mostly asymptomatic at birth, and it develops slowly for several months after birth. The posterior lens capsule begins to develop a slight bulge in early life, but it often goes unnoticed until late childhood [6]. The condition is typically diagnosed between the ages of 3 to 15 years. The primary method of diagnosis using slit-lamp microscopy is employed when the lens is not opaque. This method displays that the posterior lens capsule has a localized conical or hemispherical protrusion, which appears like an “oil drop-like” alteration [7].

Observation of the fundus through the posterior capsule changes reveals distortion and deformation of the retinal image. Conversely, the fundus image passing through the non-bulging part shows hyperopia or emmetropia, making refractive correction challenging. Occasionally, lens opacity can coincide with posterior lenticonus, and it can also develop abruptly, presenting as an all-white cataract. Posterior lenticonus shares key similarities with two congenital ocular conditions, namely, Persistent Hyperplastic Primary Vitreous (PHPV) and Posterior Polar Cataract. However, there are some notable differences. While PHPV typically lacks posterior capsule thinning and bulging, posterior lenticonus lacks concomitant microphthalmia and residual vitreous blood vessels. In the case of children with posterior polar cataracts, the posterior capsule’s appearance may resemble insect bites, but there is no posterior capsule edema. Some syndromes such as Down syndrome, Lowe syndrome, and Alport syndrome may also present similar lens issues. The patient did not exhibit any specific facial features, developmental delay or other issues that would suggest a diagnosis of Down syndrome. Absence of any glaucoma-related manifestations allows to exclude Lowe syndrome. Furthermore, the patient’s medical history did not reveal any instances of renal disease in the family, effectively ruling out the possibility of Alport syndrome.

There is currently no consensus among experts on whether surgical treatment is necessary for posterior lenticonus. A previous study has suggested that surgery is beneficial for visual acuity in such patients [8]. In general, the decision to perform surgery depends on the location of the lesion. If the lesion obscures the visual axis, surgery should be considered. This specific patient case is characterized by a localized swelling of the posterior capsule of the lens under the temporal area, which was detected through the slit lamp microscope and confirmed through various auxiliary investigations. With AS-OCT, we can see that the lesion in this patient is located far from the visual axis and does not affect the patient’s best-corrected visual acuity. It should be emphasized that compared to other ophthalmic auxiliary examinations, AS-OCT Tomography provides higher precision in observing the location of lesions. In particular, compared to UBM, AS-OCT not only shows the location of the lesion more accurately but also produces higher resolution images, and requires less skill from the operator. In this patient, free auxiliary examinations were provided in our hospital, but in general, we recommend AS-OCT Tomography as the preferred examination for this condition.

The patient’s current state of health is deemed good and as such, they have been given conservative treatment. However, it is important to note that diligent follow-up is still crucial. In the event that the cataract is advancing and causing vision impairment, timely surgery will be required. Based on this case, it can be observed that AS-OCT has advantages in diagnosing Posterior lenticonus. This technique is worth promoting for clinical use. Given that the patient’s vision meets daily requirements and there is no effect on retinal development, conservative treatment may bring more benefits to the patient. This can help avoid potential complications associated with intraocular surgery while preserving regulatory function of lens. Whether conservative treatment brings better subjective outcomes requires further investigation. However, it is indisputable that once the lesion obstructs the visual axis, surgery should be performed as soon as possible.

## Data Availability

The datasets used and/or analyzed in the course of the current study are available from the corresponding author on reasonable request.

## Abbreviations

AS-OCT: anterior segment optical coherencee
UBM: ultrasound biomicroscopy
PHPV: Persistent hyperplastic primary vitreous
